# Gynæcological Antiseptics

**Published:** 1887-03

**Authors:** E. C. Dudley

**Affiliations:** Professor of Gynæcology in the Chicago Medical College


### GYNÆCOLOGICAL ANTISEPTICS, AND OTHER MEANS OF PREVENTING PELVIC INFLAMMATIONS.

*By* E. C. DUDLEY, A.B., M.D., *Professor of Gynæcology in the Chicago Medical College.*

A CLINICAL LECTURE DELIVERED IN MERCY HOSPITAL.

*Gentlemen:*

Throughout the clinical course in gynæcology you have had opportunities of observing the manipulative features of numerous gynæcological operations, and you have therefore become familiar with operative methods. Now as the session is drawing to a close, I wish in a single lecture to present a concise resume of certain essential details which, although not distinctly operative, will yet materially aid you in securing union by first intention, and in protecting your patient against most serious, septic and inflammatory accidents. You are aware that the minor gynæcological operations are usually performed for the relief of maladies which are not often fatal, nor even always disabling, and yet these operations, if performed without proper precautions, may not only fail in union by first intention, but may even be followed by sepsis, metro-peritoneal inflammations, cellulitis, and sometimes even by death. Until very lately the statistics of minor gynæcological operations have contained so many discouraging results that the careful practitioner has often hesitated from fear, and has employed palliative and temporizing measures, however unpromising, to the exclusion of surgical measures however rational. You will recognize the importance therefore of any precautions which will render the minor operations and office manipulations comparatively free from danger. Doubtless you have already anticipated the subject of this lecture, which is Gynæcological Antiseptics. You know that the essential object of antiseptics is cleanliness —not esthetic, but surgical cleanliness. To secure and maintain surgical cleanliness many antiseptic materials have been employed, of which the one generally approved is carbolic acid. A case in one of the wards lately gave us a striking illustration of the necessity of great care in making the solution. Some of the acid not being thoroughly mixed with the water settled to the bottom of the vessel from which a douche was given, and the pure acid passed through the syringe to the vagina, and its application produced a serious burn. The addition of 10 per centum of glycerine to the carbolic acid renders it more easily soluble. The strength of your solutions should be the same for gynæcological as for other surgical purposes. A saturated solution of boracic acid or a three per centum solution of salicylic acid, unlike the carbolic, is free from caustic properties, and is at the same time an excellent antiseptic. Permanganate of potassium in solution decomposes so readily that it is unreliable for antiseptic purposes. Solutions of corrosive sublimate may be conveniently made by mixing a 10 per centum alcoholic solution with sufficient water to make the required strength, which should be from 1 in 1,000 to 1 in 10,000. The stronger solutions are adapted to the cleansing of the hands and cutaneous surfaces, and the weaker for washing the sponges during operations. Corrosive sublimate tarnishes metallic instruments and destroys their plating, but has the advantage of being odorless, of not destroying the skin, and of being a reliable germicide. The ropy, stringy mucus plug which you have so often observed in the cervix, and which is so difficult to remove, is readily coagulated by the application of a one per centum solution of corrosive sublimate to the cervical canal. This renders it easily removable, and it is also from a therapeutic standpoint an excellent application. For the washing of pus cavities you will find the peroxide of hydrogen, mixed with an equal quantity of water, a most satisfactory agent. It is especialy useful in washing the bladder in cases of cystitis.

In all gynæcological manipulations you will recognize the importance of great caution to prevent the inoculation of one patient from another. The soap, glycerine, vaseline or oil for example, which is always kept by the operator's table for the lubrication of the fingers and instruments, is frequently contaminated with gonorrhœal or other virus, and may thus become the medium of infection. Neither the fingers nor the speculum therefore should be brought in contact with any of these lubricating substances unless they have been thoroughly cleansed of all vaginal and other secretions. Many a case of vaginitis, to say nothing of syphilitic infection, has been directly traced to the carelessness of the operator in this particular. Formerly the camel's-hair pencil brush and the sponge were employed as carriers of medicinal agents in making applications to the uterus. But you will readily appreciate the difficulty of keeping them properly cleansed and their consequent unfitness for repeated use. The surgical absorbent cotton now in general use may be wound upon an applicator or stick, or may be grasped in dressing forceps, and is admirably adapted for purposes of local medication or for wiping out the genital canal. It may be used in this way a single time, and then destroyed. No special cleansing of the vagina and vulva is required for the ordinary office manipulation of these organs, except the vaginal douche, which the patient should be directed to use before coming for treatment. Interference with the interior of the uterus, however, is unsafe without more careful cleansing. Whenever the uterine cavity is to be digitally or instrumentally explored or treated, it is best to wipe out the vagina with dry absorbent cotton, and then with absorbent cotton saturated with a five per centum solution of carbolic acid with glycerine or a solution of corrosive sublimate 1 to 2,000, By this means the endometrim is protected against the entrance of septic matter, which otherwise might be carried in on the instruments from the vulva or vagina.

Preparatory to any operation on the genital tract or the abdomen, the field of operation and whatever may possibly be brought in contact therewith should be rendered surgically clean, and so maintained throughout the operation and during convalescence. These precautions apply even to the most trifling operations, which are by no means free from danger of fatal sepsis. Besides, however insignificant the proposed operation may be, it may become necessary to extend it beyond the limits of minor surgery. Frequently a seemingly minor operation in the beginning has ended accidentally or purposely in opening the abdomen or in some other serious procedure. Therefore the vulva should be thoroughly and repeatedly washed with tar soap and water, and during the week previous to operation the hot water vaginal douche should be employed twice daily each douche to contain a small quantity of castile or tar soap, except the last, to be given just before the operation, and to be a solution of corrosive sublimate 1 to 4,000, or a 2 per centum solution of carbolic acid.

The ordinary practice of simply cleansing instruments after examination with water, or soap and water, is inadequate and unsafe. Esthetic cleanliness does not absolutely destroy the virus and prevent its instrumental conveyance from one patient to another, as many unfortunate women might bear witness. It is my universal custom to secure perfect surgical cleanliness in the following manner: First, let the instruments be carefully washed in the ordinary way; then let each instrument be thoroughly wiped over with absorbent cotton saturated with equal parts of carbolic acid and glycerine. This will require the use of two strong forceps, one in the left hand to hold the instrument which is being disinfected, and the other in the right to hold the cotton mop. When the instruments have been moistened with the acid in this way and the acid has been washed off with pure water, they are ready for use. If the instruments have been unusually exposed, as for example in a pus cavity, or if they have been in contact with specific virus, it is necessary to carry the disinfection absolutely beyond question by passing them slowly through the flame of a Bunsen burner or a spirit lamp before applying the carbolic acid. If they are to be used for abdominal section first wash them with ordinary soap and water, or, still better, boil them; then use the flame, then the carbolic acid, and then place them in a pan containing water which has been boiled and filtered or distilled. This water and the adherent acid makes an excellent solution in which the instruments may remain during the operation. Cleansing and disinfection of the operator's hands and nails is imperative, not only to guard against the carrying of virus to the patient, but to prevent auto-inoculation by specific virus through some abrasion on the hand. The annoying presence of fæcal matter during an operation and its possible septic results may be avoided by giving a preparatory cathartic so early that its action may be complete before the operation. In order to render sponges free from foreign and septic matter, first thoroughly beat and wash out all the sand. This may require hours of patient labor. Then soak them over night in dilute hydrochloric acid to dissolve out calcareous matter, and after washing out the acid the sponges, which will then be much softer and more elastic, may be put away in self-sealing fruit jars containing a 5 per centum solution of carbolic acid or a 1 to 2000 corrosive sublimate solution. The solution in which the sponges are kept should be changed once a week. The boiling of sponges is an excellent antiseptic measure but it causes great shrinkage and hardening and very much lessens their absorbent properties. The silk for ligatures and sutures may be rendered thoroughly aseptic by boiling five minutes in pure carbolic acid and then for twenty minutes in a 5 per centum solution. The best Turner's braided silk treated in this way may be kept for months without injury in small wide-mouthed bottles. The braided silk is preferable to the twisted, because the latter may even be destroyed by boiling in pure acid.

The field of operation rendered aseptic in the manner already described may be kept so during the operation if attention be paid to the cleanliness of hands, sponges, instruments and other appliances. The occasional irrigation of the wound during the operation and especially while the sutures are being tightened is desirable, to secure perfect contact of the wound surfaces free from blood and other foreign bodies.

The object of the after treatment is to maintain so far as possible the aseptic condition. At the end of the operation all particles of tissue and clots of blood should be removed and the parts thoroughly cleansed by the hot water vaginal douche, which should be repeated every twelve hours until several days after the removal of the sutures. After operation on the external genitals the douche should also be given after evacuation of the bowels or bladder. Schroeder and other German operators employ constant irrigation during gynæcological operations. This requires the patient to be in the dorsal position when the operation is intra-vaginal. Professor Englemann, of St. Louis, is one of the foremost American advocates of this method, which he fully described in a communication to the American Medical Association in 1885.

TREATMENT OF SEPTIC GYNÆCOLOGICAL WOUNDS.

Certain natural conditions are favorable to the healing of wounds of the cervix and vagina. The opposite vaginal wall in contact with the wound excludes the air and acts as a compress, and the vagina makes an excellent drainage tube. But the conditions after intra-uterine operations are less favorable, because the uterine canal is at an angle of about ninety degrees to the long axis of the vagina, and the cervical portion of this canal, naturally narrow, may have become narrower from disease. Therefore, secretions accumulating in the uterine cavity may not be easily expelled by force of gravity or by uterine contractions, but on the contrary may be confined and become infectious with inflammatory and septic results. The condition simulates that of a deep abscess at the end of a long and tortuous sinus. On general principles the therapeutic indication is to cleanse the cavity and to keep it as nearly aseptic as possible by irrigation. Although this treatment is often followed by excellent results, it unfortunately is not free from grave objections, and often proves even more dangerous than the disease. Sometimes the stimulating presence of the irrigating fluid or of the canula through which it is injected causes the uterine walls to contract upon the instrument so forcibly that the return flow is impeded and the fluid passes into the fallopian tubes with excessive pain and possibly with grave inflammatory or septic results; moreover, intra-uterine injections without invasion of the fallopian tubes have many times been followed by painful uterine contractions, pelvic inflammation and death. They are therefore only to be employed when the canal throughout is sufficiently open to permit free outflow, and even then with great caution. To guard against obstruction of the outflow by contraction of the uterus upon the instrument it is necessary to use some one of the double uterine catheters, Mott's, Molesworth's, or Bozemann's for example, which have been specially devised for the purpose and which are similar in construction to Skene's double catheter for irrigation of the bladder.

The treatment of septic wounds in the uterine cavity involves some of the vexed questions in gynæcology. Intra-uterine sepsis may be indicated by the character of the discharge, by the pain, by the temperature, by the pulse, by physical signs, and by the history of the case, but it is often difficult to determine whether the disease is here confined or whether the wounded surface has not rather served as an avenue through which bacteria have passed to the pelvic cellular tissue or to the peritoneum, and there produced results which not only could not be reached by intra-uterine treatment, but which snch treatment might even aggravate. The patulous condition of the uterine canal in puerperal cases makes the organ easily and safely accessible and the treatment therefore more effective; but the most effective antisepsis in surgical gynæcology is generally prophylactic.

### OPIUM, QUININE AND ICE.

In addition to antisepsis certain other precautions against cellulitis, peritonitis and metritis should be enforced, especially in cases predisposed by a previous attack. Preparatory to operation the patient should be fortified by moderate does of opium combined with full doses of quinine, and for two or three days after the operation this should be continued and supplemented with the ice-bladder on the hypogastrium. The thin rubber ice-bladder is most convenient, but the ordinary sheet rubber such as is used by dentists for the rubber dam may be substituted by gathering up its sides and corners above the ice and tying them with strong twine. To prevent the annoying condensation of water on the outside of the rubber bag another piece of rubber or oiled silk may be wrapped about it. Experience has shown that great reliance may be placed upon opium, quinine and ice, not only for prophylaxis against inflammation but also as a remedy in the acute stage, and that the ice is much more certain in its results than the time-honored and conventional flax-seed poultice.

